# The Hippo Signaling Pathway, Reactive Oxygen Species Production, and Oxidative Stress: A Two-Way Traffic Regulation

**DOI:** 10.3390/cells13221868

**Published:** 2024-11-11

**Authors:** Bella Amanda, Rangga Pragasta, Haris Cakrasana, Arifa Mustika, Zakiyatul Faizah, Delvac Oceandy

**Affiliations:** 1 Andrology Study Program, Department of Biomedical Sciences, Faculty of Medicine, Universitas Airlangga, Surabaya 60132, Indonesia; rangga_pragasta@unisma.ac.id (R.P.); haris.cakrasana@gmail.com (H.C.); zakiyatul-f@fk.unair.ac.id (Z.F.); 2 Airlangga University Teaching Hospital, Universitas Airlangga, Surabaya 60115, Indonesia; 3 Faculty of Medicine, Universitas Islam Malang, Malang 65144, Indonesia; 4 Department of Anatomy, Histology, and Pharmacology, Faculty of Medicine, Universitas Airlangga, Surabaya 60132, Indonesia; arifa.mustika@fk.unair.ac.id; 5 Division of Cardiovascular Sciences, Faculty of Biology, Medicine and Health, University of Manchester, Manchester M13 9PT, UK; delvac.oceandy@manchester.ac.uk

**Keywords:** Hippo pathway, reactive oxygen species, oxidative stress, the mammalian Ste20-like kinases, yes-associated protein, good health and well-being

## Abstract

The Hippo signaling pathway is recognized for its significant role in cell differentiation, proliferation, survival, and tissue regeneration. Recently, the Hippo signaling pathway was also found to be associated with oxidative stress and reactive oxygen species (ROS) regulation, which are important in the regulation of cell survival. Studies indicate a correlation between components of the Hippo signaling pathway, including MST1, YAP, and TAZ, and the generation of ROS. On the other hand, ROS and oxidative stress can activate key components of the Hippo signaling pathway. For example, ROS production activates MST1, which subsequently phosphorylates FOXO3, leading to apoptotic cell death. ROS was also found to regulate YAP, in addition to MST1/2. Oxidative stress and ROS formation can impair lipids, proteins, and DNA, leading to many disorders, including aging, neurodegeneration, atherosclerosis, and diabetes. Consequently, understanding the interplay between the Hippo signaling pathway, ROS, and oxidative stress is crucial for developing future disease management strategies. This paper aimed to review the association between the Hippo signaling pathway, regulation of ROS production, and oxidative stress to provide beneficial information in understanding cell function and pathological processes.

## 1. Introduction

The Hippo signaling pathway was first discovered to play a role in the regulation of organ development in Drosophila [1]. Then, the mammalian orthologs of the Drosophila Hippo kinase were identified soon after [2,3,4,5,6,7]. The central components of the Hippo signaling pathway in mammals include the following: the mammalian Ste20-like kinases 1/2 (MST1/2), large tumor suppressor 1/2 (LATS1/2), MOB kinase activator 1 (Mob1), scaffolding protein Salvador homolog 1 (SAV1), and yes-associated protein (YAP) [8]. Over time, the other roles of the Hippo signaling pathway have been identified, such as its role in regulating cell differentiation and proliferation [9]. Hence, the Hippo signaling pathway has emerged as an interesting target to induce tissue regeneration [10]. This pathway is also critical in the regulation of apoptosis [11].

The critical role of the Hippo signaling pathway in mediating organ growth, including the brain and the neural network, has been shown in a number of studies. For example, it was shown that the Hippo signaling pathway controls neural development and differentiation [12,13]. Furthermore, deregulating this pathway increases Drosophila’s brain size [14], whereas YAP controls the cell cycle exit of the developing retina in mammals [15]. The Hippo signaling pathway is associated with some neurological diseases, such as microcephaly and hydrocephalus [16,17]. These findings indicate the importance of this pathway in brain and neural development, and it also plays a major role in the pathophysiology of some neurological diseases.

Moreover, the Hippo signaling pathway is essential in regulating cardiac development. MST1/2 and LATS1/2 deletions affect embryogenesis by increasing cardiac cell proliferation in mice. Likewise, YAP can control cardiac cell proliferation, thereby regulating heart size during embryonic development [18,19]. In adult hearts, the Hippo signaling pathway plays an important role in the regulation of cardiac hypertrophy and heart failure [20,21]. YAP and transcriptional coactivator with PDZ-binding motif (TAZ) can promote heart repair after myocardial infarction and can improve cardiac function [22,23].

The Hippo signaling pathway is involved in liver development. The deletion of MST1/2, which leads to the activation of YAP, results in liver overgrowth during embryonic development. Importantly, in adult mice, MST1/2 knockout will lead to the development of hepatocellular carcinoma [24,25]. Therefore, we may target these molecules for liver regeneration [26]. In fact, in a mouse liver damage model, the pharmacological inhibition of MST1/2 using XMU-MP1 facilitates liver regeneration [27].

In the intestines, YAP and TAZ are important during tissue regeneration and response to injury [28,29]. However, overexpression of YAP and TAZ can lead to the development of colorectal cancer [30]. This phenomenon indicates a double-sword phenomenon when targeting the Hippo signaling pathway since it induces carcinogenesis rather than tissue regeneration. Other types of cancers that have been linked with the Hippo signaling pathway are breast, lung, and pancreatic cancer [31,32,33].

Apoptosis is a key physiological process that is strongly regulated by the Hippo signaling pathway. The activation of the core components of the Hippo signaling pathway, such as MST1 and LATS1/2, is associated with the induction of apoptosis [34,35,36,37]. Some studies have shown that the Hippo signaling pathway induces apoptosis by interfering with the cell proliferation cycle [36,37], activating caspases by MST1 [38], and promoting the activation of p53 [39,40]. Furthermore, MST1 regulates apoptosis and autophagy by blocking the interaction between B-cell lymphoma 2 (Bcl-2) and B-cell lymphoma-extra large (Bcl-xL) via Beclin1 to facilitate Bcl-2-associated X protein (Bax)-stimulated apoptosis [41]. Consistent with the findings on MST1/2 and LATS1/2, previous studies have shown that YAP activation induces the expression of anti-apoptotic genes [42,43].

More recently, the Hippo signaling pathway was found to be associated with oxidative stress and reactive oxygen species (ROS) regulation, which are important in the regulation of cell survival. Low to moderate ROS levels can reduce cell apoptosis following stress stimuli and, in turn, can promote cell proliferation and survival [44,45]. Recent studies have revealed that the Hippo signaling pathway plays an essential role in the regulation of ROS production. YAP depletion increases ROS levels and, hence, induces oxidative stress-related cell death [46]. MST1/2, on the other hand, can sense excessive ROS production, thereby maintaining cellular redox homeostasis and stabilizing the key antioxidant transcription factor Nrf2 [47].

In this paper, we will review recent findings that show a close association between the Hippo signaling pathway and ROS production and oxidative stress regulation.

## 2. Hippo Signaling Pathway and Its Upstream Regulators

**Figure 1:**
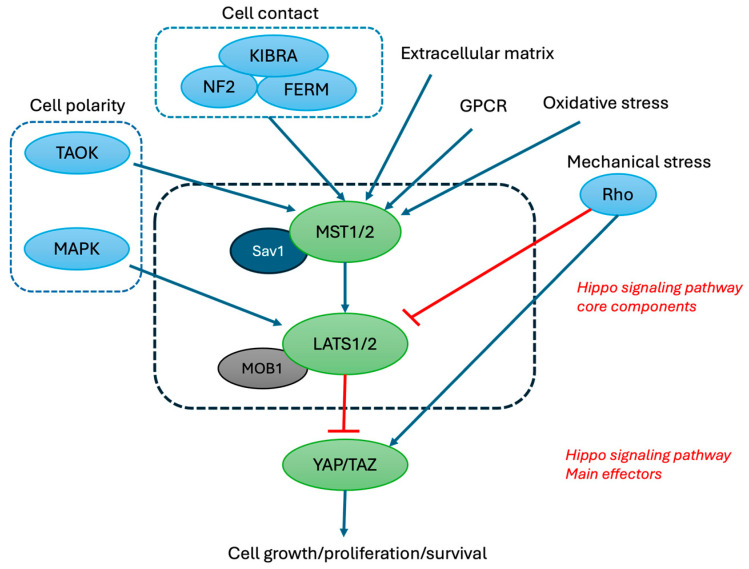
Core components of the Hippo signaling pathway and their upstream regulators. Upstream regulators of Hippo signaling pathway. Activated MST1/2 phosphorylates LATS1/2, facilitated by SAV1 and Mob1. Then, LATS1/2 will phosphorylate YAP and TAZ, leading to cytoplasmic retention and inhibition/degradation.

Approximately two decades ago, the Hippo signaling pathway was first discovered via the genetic screening of Drosophila [1,48]. In recent years, more studies have provided information that the Hippo signaling pathway has broader roles than just controlling organ size during embryonic development [10]. Figure 1 depicts the core regulatory mechanism of the Hippo signaling pathway. When activated, MST1/2 phosphorylates SAV1 and Mob1, which will facilitate the recruitment and phosphorylation of LATS1/2 to the molecular complex. Activated LATS1/2 will phosphorylate YAP and TAZ, leading to cytoplasmic retention and degradation [49]. Conversely, reduced MST1/2 activation results in the activation of YAP and TAZ, which leads to nuclear translocation and the induction of gene expression by binding to the TEAD transcription factor family [27,50,51]. This will result in the induction of genes involved in cell proliferation, migration, and survival [50,52,53].

The core components of the Hippo signaling pathway are regulated by several different upstream signals. They can be activated by cell-to-cell contact through the FERM domain proteins, Neurofibromatosis type-2 (NF2), and the WW and C2 domain-containing protein renal and brain expressed protein (KIBRA) [54,55,56]. Changes in cell polarity can also activate the Hippo signaling pathway through TAOKs by phosphorylating MST1/2 and MAPK by phosphorylating LATS1/2 [57,58]. Furthermore, mechanical stress induces cytoskeleton reorganization, which leads to the activation of the Hippo signaling pathway, primarily through the modulation of LATS1/2 activity by Rho GTPases [59]. Changes to the extracellular matrix, GPCRs, and oxidative stress are additional potential factors that influence the Hippo signaling pathway [60,61].

Cell-to-cell contact modulates the interaction of the apical membrane-associated FERM-domain protein NF2 and KIBRA (also known as WWC1) to activate the core components of the Hippo signaling pathway. FERM domain proteins, including NF2, have a role in the regulation of cellular architecture [55,62]. NF2, which has mutations linked to neurofibromatosis type II, suppresses tumors and activates the Hippo signaling pathway components. NF2 and KIBRA work synergistically to promote the Hippo signaling pathway, with FERM domain proteins facilitating their coordination [62,63].

GPCRs are a family of receptors in the cell membrane that detect extracellular substances (ligands) and can activate intracellular responses. The Hippo signaling pathway is regulated by GPCR ligands via different methods. The type of intracellular messengers downstream of the G proteins, activated by the ligand’s binding to GPCRs, determines the downstream effects after YAP and TAZ activation [49]. By inhibiting the LATS kinase, estrogen activates YAP/TAZ through the GPCR. This process involves the modulation of Gαq/11, PLCβ, PKC, Rho GTPases, and ROCK activities and is important in the development of breast cancer [64]. Other types of cancer, such as colon, ovarian, prostate, lung, and pancreatic, as well as hepatocellular carcinoma (HCC) and melanoma, are associated with GPCR-induced YAP activation [65].

Extracellular matrix (ECM) and cell polarity are the other upstream regulators of the Hippo signaling pathway. ECM stiffness inactivates the Hippo signaling pathway, which will eventually lead to a higher expression of YAP. Some studies have shown that HCC expresses high agrin and fibrillary collagen levels. This can stiffen the ECM and eventually increase the amount of nuclear YAP/TAZ [66]. Another example of regulation by ECM was the disruption of adherens junctions (AJs) or Scribble, which resulted in the deregulation of the Hippo signaling pathway and the overexpression of YAP/TAZ. In Drosophila, the knockdown of AJs causes reduced Yki activity and cell death [67].

Mechanical stress, oxidative stress, and DNA damage can stimulate the Hippo signaling pathway. Mechanical stresses such as stretching, strain, compression, and pressure might affect the mechanical properties of extracellular matrices. Based on previous studies, changes in the extracellular matrix affect the expression and activation of the Hippo signaling pathway components. Mechanical stresses can modulate the expression of YAP/TAZ via Rho GTPase activity and GPCR signaling [59].

In keeping with those findings, some studies have shown that MST1/2 is activated by hydrogen peroxide (H_2_O_2_) in the cellular oxidative stress response [68,69]. Interestingly, the stress response due to the stimulation of the Hippo signaling pathway might involve other proteins or pathways, such as FOXO1, AMPK, and TOR [49].

## 3. Regulation of ROS by Hippo Signaling Pathway Components

ROS are part of the cellular defense system and are produced as a response to external stimuli (ultraviolet and ionizing radiation, pollutants, and heavy metals), xenobiotics (i.e., antiblastic drugs), growth factors, stress stimuli, the presence of cytokines, and bacterial invasion [70]. ROS act as signaling molecules for the oxidation of target proteins and for the regulation of the on–off switch proteins in the signaling pathways [71,72]. Mitochondria primarily produce ROS through oxidative metabolism [73]. A small increase in ROS induces proliferation, differentiation, migration, and angiogenesis [74]. However, a high ROS level can overwhelm the cellular antioxidant defense system, elicit cellular oxidative stress, and induce aging and cell death [71,75].

Studies on MST1 involvement in ROS production showed a correlation between the Hippo signaling pathway and ROS [76,77]. Overexpression of MST1 induces cell death and apoptosis via the activation of caspases 1, 3, and 7. It is interesting that in some situations, the expression of MST1 is linked to that of caspase-1, which is an important part of normal inflammasome-mediated pyroptosis (ROS production). This provides evidence of the possible involvement of MST1 in ROS production [38].

In addition to MST1, YAP and TAZ are also involved in ROS production. White et al. performed a study using NF2-deficient tumor cells. They showed that YAP/TAZ depletion increases mitochondrial respiration and ROS production, resulting in the oxidative stress-induced death of tumor cells [46].

In contrast, studies on cardiac cells have shown that YAP expression is involved in neutralizing the damaging effects of excessive ROS production. Shao et al. have reported that YAP expression protects cardiomyocytes against peroxide-induced cell death [61]. The mechanism likely involves the activation of transcriptional co-activator FOXO1. The YAP-FOXO1 interaction leads to the induction of antioxidant gene expression, such as catalase and MnSOD [61]. This will produce a protective effect by reducing the accumulation of ROS in the heart.

**Figure 2:**
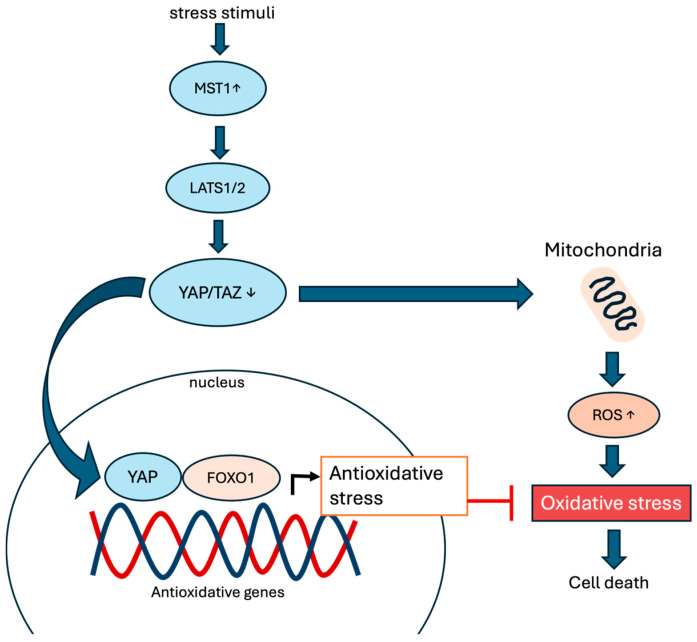
Hippo signaling pathway in ROS production. Stress stimuli activate MST1/2 and phosphorylate LATS1/2. LATS1/2 will then phosphorylate YAP and TAZ, leading to increased mitochondrial respiration, elevated ROS, oxidative stress, and eventually cell death.

Research using a liver damage model, in accordance with the abovementioned studies on the mouse heart, has shown the role of YAP in controlling oxidative stress. The activation of YAP through ischemia protects the liver from damage caused by ischemia and reperfusion. The ability of YAP to induce the expression of antioxidative genes, thereby reducing the detrimental effects of excessive ROS production (Figure 2), is responsible for this [63,78].

## 4. Mechanisms of Hippo Signaling Pathway Activation by ROS

An elevated level of intracellular ROS, including hydrogen peroxide (H_2_O_2_), superoxide (O_2_), and hydroxyl (OH)-free radicals, causes oxidative stress [70]. The role of ROS in pathological conditions is complex. For example, in the development of cancer, moderate ROS levels can promote cell proliferation and migration, thereby contributing to tumor development [79]. In contrast, excessive ROS or persistent oxidative stress can cause oxidative damage to lipids, proteins, and DNA, leading to apoptosis and senescence, which subsequently prevent tumor development [80,81,82].

Oxidative stress can activate the Hippo signaling pathway via many methods, affecting its core components, particularly MST1/2 and LATS1/2. MST1 is activated under oxidative stress, especially in the presence of hydrogen peroxide. H_2_O_2_ primarily influences the Hippo signaling pathway by regulating the E3 ubiquitin ligase NEDD4. NEDD4 has been associated with the regulation of the Hippo signaling pathway in reaction to oxidative stress [83]. Studies show that a low dose of H_2_O_2_ enhances the expression of NEDD4. The elevation of NEDD4 and ubiquitin E3 ligase affects the degradation of LATS1/2 and activates YAP/TAZ, promoting proliferation [84,85]. A larger dose of H_2_O_2_ (>200 µM) induces oxidative stress and death by activating the Hippo signaling system [83,86]. Furthermore, MST1 is specifically activated by H_2_O_2_, resulting in its participation in pro-apoptotic signaling pathways. The activation of MST1 in response to oxidative stress is facilitated by its interaction with peroxiredoxin-1 (Prdx1), an antioxidant enzyme that decreases H_2_O_2_ levels. Excessive H_2_O_2_ levels result in the inactivation of Prdx1, causing its oligomerization and the subsequent activation of MST1, which promotes apoptosis [87]. In cultured astrocytes, treatment with H_2_O_2_ activates MST1, which eventually causes cell death [88]. Increasing H_2_O_2_ levels also activates Neurofibromin 2 (NF2), which results in enhanced phosphorylation of MST1 and YAP, supporting the concept that NF2 functions as a modulator of oxidative stress responses via the Hippo pathway [89]. Research has shown that cells lacking NF2 exhibit elevated oxidative stress, which promotes cancer development and the onset of diseases such as neurofibromatosis type 2 [90,91]. 

**Figure 3:**
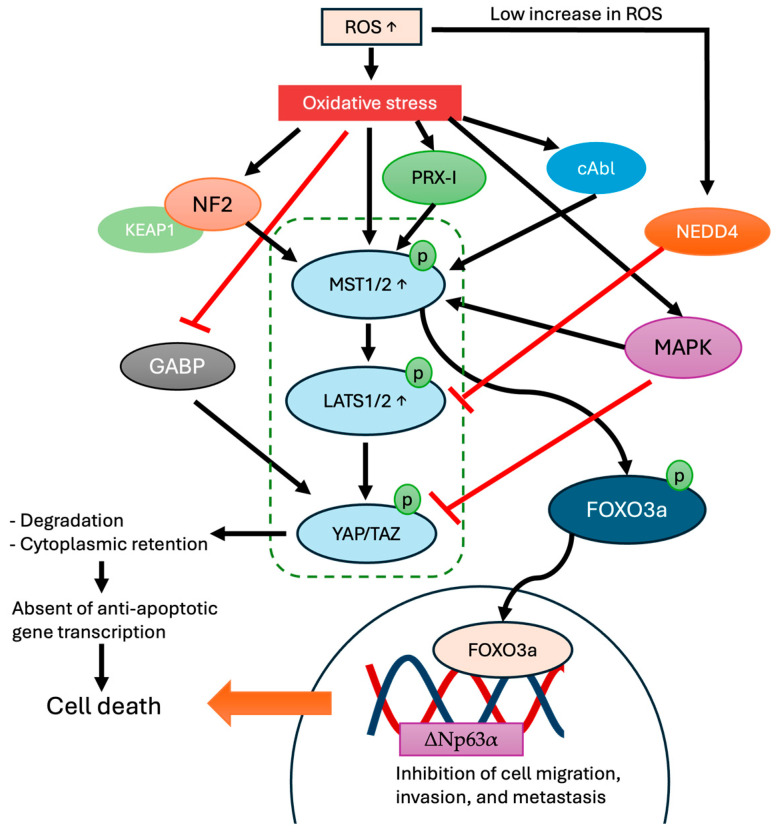
Oxidative stress activates Hippo signaling pathway. Oxidative stress modulates NF2, PRX-I, cAbl, and MAPK to phosphorylate MST1/2. Simultaneously, MAPK inhibits YAP translocation. Meanwhile, GABP is inhibited by the presence of oxidative stress, which will inhibit expression of YAP/TAZ. All of these lead to the nuclear entry inhibition of YAP, which is responsible for the production of pro-proliferative and anti-apoptosis proteins. In addition, phosphorylated MST1 induces phosphorylation of FOXO3a and activates transcription of ΔNp63α, which inhibits cell migration, invasion, and metastasis. Concurrently, mild elevations in ROS activate NEDD4, which subsequently ubiquitinates LATS, thereby inhibiting its function.

To further control responses to oxidative stress, the Hippo signaling pathway interacts with transcription factors known as Forkhead box O (FOXO). FOXO1 and YAP work together to control antioxidant gene expression when ROS stimulate its nuclear translocation [61]. Through this interaction, the Hippo signaling pathway helps in the cellular defense mechanism against ROS (Figure 3). Using different cellular models, Wang et al. revealed that ROS can induce MST1/2 activation [92]. Then, MST1/2 causes FOXO3a phosphorylation, resulting in a higher expression of ΔNp63α, which is a direct transcriptional target of FOXO3a. This mechanism will subsequently reduce cell migration. Interestingly, this modulatory pathway involving ROS-MST1/2—FOXO3a and ΔNp63α—is independent of YAP activation, which supports the idea of non-canonical Hippo activation by ROS [92].

Other studies have shown the association between Hippo signaling pathway activation and ROS-dependent pathways. For instance, a study found that curcumin-induced ROS production in melanoma cells activates MST1. When MST1 kinase was activated, it phosphorylated FOXO3 and increased Bim expression through FOXO3 [93]. This caused cells to die by apoptosis. It was shown in another study using primary mammalian neuronal cells that oxidative stress might activate MST1 through c-Abl tyrosine kinase. MST1 is phosphorylated by c-Abl at Tyr433, leading to the binding of MST1-FOXO3, which subsequently causes oxidative stress-induced neuronal cell death [94,95].

The p38 mitogen-activated protein kinase (MAPK) pathway has also been identified as an upstream regulator, suggesting that several signaling pathways can affect Hippo signaling activity [58]. Oxidative stress has effects on this pathway; for example, high ROS levels activate p38 MAPK, which, in turn, phosphorylates MST1/2 and inhibits YAP nuclear translocation that mediates cell differentiation, death, and inflammatory responses [96,97].

ROS and oxidative stress regulate YAP in addition to MST1/2. Zhou et al. assessed the involvement of YAP in the oxidative stress response [98]. The liver’s GA-binding protein (GABP) regulates the expression of YAP, according to the results [99]. Importantly, YAP expression is decreased when GABP activity is inhibited by oxidative stress, indicating that the reduction in YAP contributes to a diminished response to oxidative stress [98].

Oxidative stress and ROS accumulation can damage lipids, proteins, and DNA and induce various diseases, such as aging, neurodegeneration, atherosclerosis, and diabetes [78,100]. Table 1 summarizes the involvement of Hippo signaling pathway components in ROS production and oxidative stress regulation. Among the members of the Hippo signaling pathway, MST1/2 and YAP can be the main proteins associated with ROS/oxidative stress regulation. To date, more studies have focused on therapeutic strategies targeting the proteins of the Hippo signaling pathway. Researchers found that XMU-MP1, an MST1/2 inhibitor, reduces oxidative stress in cardiomyocytes [101]. TT-10 is another compound that could affect the Hippo signaling pathway (YAP/TAZ activator). Research has shown that TT-10 can effectively decrease the production of ROS caused by myocardial infarction [102]. Therefore, pharmacological targeting of the Hippo signaling pathway is a prospective strategy for modulating ROS production and oxidative stress regulation and can be useful for therapeutic purposes. For example, it can improve cell survival, induce cell regeneration, or even control cancer cell growth.

## 5. Possible Future Applications

It can be speculated that, based on existing knowledge, oxidative stress plays a role in several diseases, including cancer and cardiovascular, neurodegenerative, and kidney diseases. If we can control oxidative stress through the Hippo signaling pathway, it could lead to novel therapeutic strategies and improve disease outcomes.

In cancer treatment, targeting the Hippo signaling pathway has become a feasible approach, particularly in malignancies marked by dysregulated YAP/TAZ activity. Inhibiting the development of the YAP/TAZ-TEAD complex with drugs such as verteporfin has demonstrated potential in preclinical models, indicating that enhancing the Hippo signaling pathway can be used as an anti-tumor strategy [105]. The involvement of deubiquitinating enzymes in regulating TAZ expression underscores an additional regulatory mechanism that may be leveraged for therapeutic strategies in gastric cancer and other malignancies [106].

The Hippo signaling pathway’s role in oxidative stress responses presents opportunities for addressing disorders like cardiovascular disease, where increased activation of the Hippo signaling pathway affects cardiac function [85]. Inhibiting the route could enhance cardiomyocyte survival and facilitate regeneration in the context of oxidative stress-induced damage [107]. Moreover, the regulatory function of the system in stem cell biology indicates that altering the Hippo signaling pathway may improve stem cell therapy for numerous degenerative diseases [10].

Modulating the Hippo signaling pathway also offers fascinating opportunities for disorders linked to oxidative stress. A possible application involves improving tissue regeneration and repair processes. Research indicates that the therapeutic inhibition of the Hippo signaling pathway or the enhancement of YAP activity improves the efficacy of tissue regeneration post-injury [108]. This is especially relevant in situations when oxidative stress compromises cellular function and viability. Precise pharmaceutical intervention with perfect timing is necessary to temporarily activate YAP, as its continuous activation is associated with oncogenesis [108].

It is essential to acknowledge that the Hippo signaling pathway has varying effects on different organs. In the heart, the activation of the Hippo signaling pathway can promote cardiomyocyte apoptosis and exacerbate chemotherapy-induced cardiotoxicity [61,85,109]. This indicates that although the Hippo signaling pathway may function as a tumor suppressor in cancer, its activation in heart tissue might result in negative outcomes, emphasizing its context-dependent function [110]. Conversely, in the liver, the Hippo signaling pathway is essential for modulating hepatocyte proliferation and inhibiting carcinogenesis [111].

However, various problems must be addressed in order for this research to be applied in the future. One key problem is the possibility of off-target effects. Off-target effects can cause unwanted mutations that potentially result in undesirable effects, such as oncogenesis or other genotoxic results. Furthermore, given the complexities of the Hippo signaling pathway linkages with other pathways, dissecting these relationships requires a rigorous and systematic approach, as alterations in one route can have cascade effects on others [112]. Finally, transferring findings from animal models to human physiology remains a major challenge, as regulatory mechanisms might vary greatly between species. Addressing these problems will be critical for improving our understanding of the Hippo signaling pathway’s involvement in health and disease, as well as creating accurate therapeutic options.

## 6. Conclusions

ROS and oxidative stress play major roles in the regulation of several physiological and pathological processes, including apoptosis and cell survival. The Hippo signaling pathway is a highly conserved molecular signaling pathway that regulates cell growth, survival, and apoptosis. Recent studies have shown an association between the Hippo signaling pathway, ROS production, and oxidative stress regulation. Some members of the Hippo signaling pathway are involved in ROS production. Further, ROS and oxidative stress induce the activation of some key components of the Hippo signaling pathway.

The association between the Hippo signaling pathway and ROS production and oxidative stress regulation provides new insights into the beneficial effects of modulating ROS/oxidative stress. The identification of novel pharmacological compounds that can specifically inhibit or activate the components of the Hippo signaling pathway provides new opportunities to identify novel therapeutic approaches to manage diseases correlated with ROS and oxidative stress.

## Figures and Tables

**Table 1:** Association between the Hippo signaling pathway and ROS/oxidative stress.

Author	Results
Xiao et al., 2011 [95]	c-Abl-MST1 signaling mediates oxidative stress-induced transcriptional activation of FOXO3 and neuronal cell death.
Morinaka et al., 2011 [87]	PRX-I induces cell death in response to elevated oxidative stress via activating MST1.
Yu et al., 2013 [93]	Activated MST1 induces JNK activation, Foxo3a nuclear accumulation, and Bim-1 expression to promote melanoma cell apoptosis.
Shao et al., 2014 [61]	YAP deletion increases ROS levels in the mouse heart. The YAP-FoxO1 complex mediates the expression of the antioxidant gene.
Lee et al., 2014 [88]	cAbl and MST1 simultaneously activated under oxidative stress.
Rajesh et al., 2016 [84]	ATF4 enhances LATS1 stability by inhibiting certain ubiquitin ligases (NEDD4.2 and WWP1), which phosphorylate YAP, resulting in its inactivation, suppression of antioxidant gene expression, and promotion of cell death.
Hamon et al., 2017 [103]	The YAP/TEAD complex, comprising Ctgf and Cyr61, is increased in response to oxidative stress during retinal degeneration.
White et al., 2019 [46]	YAP/TAZ depletion increases mitochondrial respiration and ROS levels.
Cui et al., 2019 [38]	MST1 promotes cell death via caspase-1-induced pyroptosis in pancreatic cells.
Wang et al., 2019 [92]	Oxidative stress signals the Hippo signaling pathway to start its downstream process by inducing the phosphorylation of JNK that upregulates ΔNp63α expression (a direct transcriptional target of FOXO3a), resulting in the inhibition of cell migration independent of YAP.
Zhou et al., 2019 [72]	YAP deletion promotes lung cancer cell death via the JNK-MIEF1 pathway.
Liu et al., 2019 [78]	YAP activation caused by ischemia in experimental liver injury promotes the expression of antioxidative genes, which has a protective effect against the liver ischemia–reperfusion injury model.
Nakatani et al., 2021 [82]	Increased ROS levels activated LATS1/2 kinases, resulting in the phosphorylation of YAP/TAZ, which caused their exclusion from the nucleus and subsequent proteasomal destruction.
Zhou et al., 2022 [81]	H_2_O_2_ stimulated ROS-mediated activation of Hippo signaling in rNP cells by promoting the phosphorylation of Mst1/2, Lats1/2, Yap, and Taz proteins, while concurrently downregulating the expression of Yap and Taz in rNP cells.
Li et al., 2023 [104]	Chaetocin induces ROS buildup and has anti-ESCC action via activating the Hippo signaling pathway, as seen by drastically reduced levels of MST1/2, MOB1, LATS1, and YAP following chaetocin treatment of ESCC cell lines.
Zou et al., 2023 [85]	In Dox-treated cardiomyocytes, NEDD4-2 is increased, resulting in the activation of the Hippo pathway (phosphorylation of MST1, LATS2, and YAP), which contributes to mitochondrial dysfunction and oxidative stress.
Kwon et al., 2024 [75]	Mitochondrial stress promoted YAP/TAZ dephosphorylation, nuclear accumulation, and target gene transcription. RhoA oxidation by mitochondrial superoxide resulted in LATS-dependent and -independent activation of YAP/TAZ.

## Data Availability

Not applicable.
